# Acute endocrine, immune, and muscle damage responses following a 2,000-m rowing time trial in elite male athletes 

**DOI:** 10.3389/fphys.2025.1712471

**Published:** 2025-12-09

**Authors:** Soo-Min Ha, Min-Seong Ha, Minchul Lee

**Affiliations:** 1 Department of Physical Education, Pusan National University, Busan, Republic of Korea; 2 Laboratory of Sports Conditioning: Nutrition, Biochemistry, and Neuroscience, Department of Sport Science, College of Arts and Sports, University of Seoul, Seoul, Republic of Korea; 3 Department of Sports Medicine, College of Health Science, CHA University, Pocheon-si, Gyeonggi-do, Republic of Korea

**Keywords:** rowing, stress hormones, immune response, muscle damage, recovery, elite atletes

## Abstract

**Background:**

Rowing is a whole-body sport characterized by high-intensity efforts that require simultaneous aerobic and anaerobic energy provision. Although endurance physiology is well documented, the acute systemic responses to competitive rowing remain insufficiently understood. The present study examined endocrine stress responses, leukocyte redistribution, and muscle damage dynamics following a 2,000-m rowing time trial in elite male athletes.

**Methods:**

Twenty national-level rowers (age: 20.4 ± 1.2 years; height: 180.0 ± 4.3 cm; body mass: 80.6 ± 8.7 kg) completed a standardized 2,000-m ergometer test. Venous blood samples were collected at baseline (PRE), immediately post-exercise (POST), and 30 min after exercise cessation (REC30). Serum cortisol was quantified by radioimmunoassay, plasma catecholamines by high-performance liquid chromatography, leukocyte subpopulations by flow cytometry, and muscle damage markers (creatine kinase [CK], lactate dehydrogenase [LDH]) by enzymatic analysis. Normality was evaluated, and repeated-measures ANOVA or Friedman tests were applied with Bonferroni-adjusted post-hoc comparisons.

**Results:**

Mean performance time was 6:49.6 ± 14.7 min, corresponding to a mean power output of 328.4 ± 34.1 W. Cortisol, epinephrine, and norepinephrine increased markedly at POST (p < 0.001) and remained elevated at REC30. Total white blood cells, neutrophils, monocytes, eosinophils, and basophils peaked at POST and declined toward baseline by REC30. Lymphocytes rose at POST but decreased below baseline at REC30 (p < 0.001), and the neutrophil-to-lymphocyte ratio exhibited a biphasic pattern. CK and LDH increased acutely at POST and partially regressed at REC30, remaining above baseline.

**Discussion:**

These findings indicate that maximal rowing performance elicits pronounced endocrine activation, transient immune redistribution, and acute muscle damage. Tailored recovery strategies may be necessary to counteract immune suppression and support sustained performance in elite rowers.

## 1 Introduction

Rowing is a globally competitive Olympic discipline that uniquely combines aerobic endurance with high-intensity anaerobic power demands. The standard 2,000-m race is completed in 5.5–7 min, requiring athletes to maintain near-maximal oxygen consumption (VO_2_max) while generating substantial power across repeated full-body stroke cycles (Kaczmarczyk et al., 2025). Unlike predominantly endurance-based sports such as marathon running or cycling, rowing requires integrated cardiovascular, metabolic, and neuromuscular adaptations. Performance depends on technical efficiency and the capacity to sustain high rates of work against increasing metabolic acidosis (Gomes et al., 2020; Bizjak et al., 2021).

The acute physiological stress induced by 2,000-m rowing has been characterized as a “hybrid” load, the effort simultaneously taxes both anaerobic glycolytic pathways and aerobic oxidative metabolism of 2,000-m rowing performance. It eliciting rapid lactate accumulation, elevated ventilatory strain, and profound cardiovascular activation (Ferreira et al., 2016). These demands are accompanied by endocrine adjustments, notably activation of the hypothalamic-pituitary-adrenal (HPA) axis and the sympathetic-adrenal-medullary system, which increase circulating cortisol, epinephrine, and norepinephrine to mobilize substrates, regulate hemodynamics, and support performance (Haller et al., 2023; Yi et al., 2025). While these stress hormones facilitate acute adaptation, elevated or dysregulated responses may impair recovery, suppress immune function, and contribute to overtraining syndrome (Gong et al., 2015; Peake et al., 2017).

Immune responses to exercise are complex and transient. Acute strenuous exercise induces leukocytosis primarily through catecholamine-mediated demarginating of neutrophils and lymphocytes (Gustafson et al., 2017; Markov et al., 2023). During recovery, lymphocyte counts often fall below baseline, creating a temporary “open window” of immune suppression (Shinkai et al., 1997; Campbell and Turner, 2018; Nanavati et al., 2022). This period may increase susceptibility to upper respiratory tract infections, particularly in athletes engaged in high-frequency or high-volume training (Oray et al., 2016; Simpson et al., 2021). Monitoring leukocyte subsets and ratios, such as the neutrophil-to-lymphocyte (N/L) and monocyte-to-lymphocyte (M/L) indices, provides a practical biomarker-based approach for quantifying systemic stress and immune status in athletes (Gustafson et al., 2017; Markov et al., 2023). Based on established exercise-induced leukocyte trafficking responses, we hypothesized that the 2,000-m rowing time trial would elicit a transient lymphocytopenia during early recovery, accompanied by increases in neutrophil counts and shifts in leukocyte ratios (e.g., N/L, M/L), reflecting acute immunological stress and cell redistribution. While these physiological expectations are directional, all statistical analyses were conducted using two-sided tests, consistent with standard analytical approaches in exercise immunology.

In addition, skeletal muscle damage is a common consequence of repeated high-intensity rowing strokes, which combine concentric and eccentric contractions of large muscle groups under high loads (Ferreira et al., 2016; Yi et al., 2025). Circulating markers such as creatine kinase (CK) and lactate dehydrogenase (LDH) reflect sarcolemmal disruption and metabolic stress (Gomes et al., 2020). Elevated levels are widely used to monitor exercise-induced muscle damage, but their kinetics differ depending on sport modality, intensity, and recovery interventions (Haller et al., 2023). In rowing, where training involves daily exposure to high mechanical and metabolic strain, understanding acute changes in muscle damage markers is essential for preventing maladaptation and overuse injury (Markov et al., 2023).

Despite growing research in exercise immunology and endocrinology, relatively few studies have comprehensively examined the integrated endocrine, immune, and muscle damage responses to 2,000-m rowing performance in elite athletes (Gustafson et al., 2017). Given the central role of this distance in international competition, clarifying these physiological responses can inform optimization of performance, recovery, and long-term athlete health. Therefore, the present study examined acute changes in cortisol, catecholamines, leukocyte subsets, and muscle damage markers in national-level male rowers before, immediately after, and 30 min after a maximal 2,000-m rowing ergometer trial. We hypothesized that the trial would elicit significant increases in stress hormones, leukocyte redistribution consistent with transient immunosuppression, and elevations in CK and LDH, reflecting the metabolic and mechanical demands of rowing competition.

## 2 Materials and methods

### 2.1 Participants

Twenty elite male rowers with a minimum of 4 years of competitive experience and at least one medal performance in a national championship participated in this study, meeting the definition of national-level athletes as defined by previous study (McKay et al., 2022). Sample size was calculated using G*Power 3.1.9.7 for repeated-measures ANOVA (within factors), with an α level of 0.05, statistical power of 0.80, effect size f = 0.25, three measurements, and a correlation among repeated measures of r = 0.65, indicating a required minimum sample of 20 participants. The athletes had a mean age of 20.37 ± 1.19 years; rowing career length 5.45 ± 1.43 years; height 179.96 ± 4.33 cm; body mass 80.57 ± 8.73 kg; and body mass index (BMI) 24.86 ± 2.36 kg/m^2^ 7 athletes (35.0%) competed in the lightweight class, and 13 (65.0%) were classified as heavyweight rowers. Descriptive characteristics are presented in Table 1.

**TABLE 1:** Physical characteristics of participants.

Variable	All (n = 20)
Age (y)	20.37 ± 1.19
Sport age (training years, y)	5.50 ± 1.36
Weight category, n (%): Lightweight; heavyweight	Lightweight 7 (35.0); heavyweight 13 (65.0)
Height (cm)	179.96 ± 4.33
Body mass (kg)	80.57 ± 8.73
BMI (kg·m^2^)	24.86 ± 2.36
Skeletal muscle mass (kg)	39.55 ± 3.58
Percentage bodyfat (%)	14.24 ± 3.74

Values are mean ± SD, unless otherwise stated; categorical variables are n (%).

Inclusion criteria required that participants be free from musculoskeletal or neurological disorders and engaged in structured training for ≥6 days per week, ≥5 h per day. Athletes failing to meet these criteria were excluded. Participants’ health status was confirmed using: (1) a self-reported health questionnaire, (2) resting body temperature (<37.5 °C), and (3) the absence of clinical symptoms such as cough, sore throat, nasal congestion, dyspnea, or myalgia. Individuals reporting signs of infection, recent medication use (e.g., anti-inflammatory agents, antibiotics), or vaccination within the previous 2 weeks were excluded. All procedures were approved by the Institutional Review Board of Pusan National University (Approval No. PNU IRB/2016_24_HR). Written informed consent was obtained from all participants in accordance with the Declaration of Helsinki.

### 2.2 Measurements and methods

#### 2.2.1 Body composition assessment

Body height was measured using a stadiometer (BSM-370, InBody Co., Ltd., Seoul, Korea) and body mass, skeletal muscle mass, and body fat percentage were determined using a multi-frequency bioelectrical impedance analyzer (InBody 770, InBody Co., Ltd., Seoul, Korea), applying a clothing offset correction of −0.6 kg as per the device’s standard calibration procedure with participants wearing light clothing. To minimize fluid balance effects, participants refrained from food and fluid intake for 2 h prior to testing and were asked to void their bladder immediately before measurement.

#### 2.2.2 2,000-m rowing ergometer test

Performance testing was conducted under standardized laboratory conditions (temperature 22 °C–24 °C, relative humidity 40%–60%). All measurements were conducted between 08:00 and 11:00 to minimize diurnal variation. A rowing ergometer (Concept2, Model E, PM4, United States) with a drag factor fixed at 130 was used. Following baseline venous sampling (PRE), all athletes completed a 10-min standardized warm-up on the rowing ergometer, consisting of (1) 5 min of continuous rowing at <2 mM lactate intensity (∼50% VO_2_max), (2) three sets of 10–15 progressive strokes increasing stroke power up to race pace, and (3) 1–2 min of seated passive rest before the time trial. This protocol was adapted from previously validated short warm-up procedures in elite rowing (Mujika et al., 2012) to minimize fatigue while ensuring neuromuscular readiness. And then rested seated for 3 min before initiating the trial. Each participant performed a 2,000-m time trial with instructions to maintain maximal sustainable effort. During the 2000-m test, athletes received standardized verbal encouragement every 250–300 m from the same investigator (e.g., “Keep your pace,” “500 m left, stay strong,” “Push through”) to ensure a consistent motivational stimulus across participants. Blood was collected immediately post-exercise (POST) and at 30 min of recovery (REC30). Performance outcomes—including total time (mm: ss.s), average split (s·500 m^-1^), mean power (W), relative power (W·kg^-1^), average stroke rate (spm), and stroke count—were obtained directly from the performance monitor (PM4).

Although a separate control trial was not conducted due to practical constraints in recruiting elite athletes, previous research has demonstrated that acute cortisol responses to maximal exercise are highly reproducible under standardized conditions (Leal et al., 2019). Therefore, hormonal responses observed in this study are considered methodologically robust.

#### 2.2.3 Blood biomarker analysis

Venous blood was drawn from the antecubital vein at PRE, POST, and REC30. Samples were processed using biomarker-specific protocols:

Hormones: Cortisol was quantified from serum after clotting (20–30 min at room temperature) and centrifugation (4 °C, 2,000 g, 10 min). Serum cortisol was quantified via radioimmunoassay (RIA) with a commercial kit (CORTISOL RIA CT, AMP, Germany) and a γ-counter (Cobra 5,010 Quantum, Packard Instrument Co., United States). Catecholamines (epinephrine, norepinephrine) were analyzed from EDTA plasma collected on ice, centrifuged (4 °C, 2,000 g, 10 min), stored at −80 °C, and batch-assayed using HPLC (Alliance system, Waters, United States) with the Plasma Catecholamine Kit (Chromsystems, Germany).

Immune Cell Profile: Complete blood counts with differential leukocyte analysis (neutrophils, lymphocytes, monocytes, eosinophils, basophils) were performed within 2 h using a flow cytometry-based hematology analyzer (XN-9000, Sysmex, Japan) with manufacturer-recommended reagents (CELLPACK DCL, SULFOLYSER, Lysercell WNR/WDF, CELLPACK DFL, Fluorocell). Absolute counts were obtained, and the neutrophil-to-lymphocyte ratio (N/L) and monocyte-to-lymphocyte ratio (M/L) were calculated.

Muscle Damage Markers: CK and LDH were measured in serum using an enzymatic kinetic assay with an automated analyzer (Modular Analytics P, Roche, Germany).

### 2.3 Statistical analysis

All statistical analyses were performed using SPSS version 25.0 (IBM Corp., Armonk, NY, United States). Data are presented as mean ± SD. Normality was tested using the Shapiro–Wilk test. Variables violating normality were log-transformed, and transformed values were used for parametric analyses. When normality was not achieved even after transformation (e.g., N/L ratio), non-parametric analyses were additionally performed. Repeated-measures comparisons across PRE, POST, and REC30 were conducted using one-way rm-ANOVA. Sphericity was assessed using Mauchly’s test, with Greenhouse–Geisser correction applied when violated. Significant main effects were followed by Bonferroni-adjusted pairwise comparisons (α = 0.0167). Effect sizes were expressed as partial eta squared (η^2^p). For variables remaining non-normal, the Friedman test and Wilcoxon signed-rank tests with Bonferroni correction were used. Statistical significance was set at p < 0.05.

## 3 Results

### 3.1 2,000-m rowing performance

The performance profile of the 2,000-m rowing ergometer test is presented in Table 2. The mean completion time was 6:49.6 ± 14.7 s (mm:ss.s), corresponding to an average split of 102.39 ± 3.67 s·500 m^-1^. Mean absolute power output was 328.41 ± 34.1 W, with a relative power of 4.11 ± 0.53 W kg^-1^. The average stroke rate was 31.42 ± 1.98 strokes·min^-1^, and the mean stroke distance was 9.37 ± 0.60 m·stroke^−1^ (Table 2).

**TABLE 2:** Rowing performance metrics in the 2,000-m time trial (n = 20).

Variable	All (n = 20)
2000-m time trial (mm:ss.s)	6:49.6 ± 14.7
Average split (s·500 m^-1^)	102.39 ± 3.67
Mean power (W)	328.41 ± 34.1
Relative power (W·kg^-1^)	4.11 ± 0.53
Average stroke rate (strokes·min^-1^; spm)	31.42 ± 1.98
Distance per stroke (m/stroke)	9.37 ± 0.60

Values are mean ± SD.

### 3.2 Stress hormones

**FIGURE 1:**
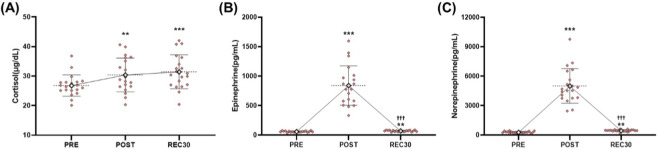
Stress hormone responses to a 2000-m rowing race. Individual values (pink diamonds) and group mean ± SD (white diamonds) are shown for **(A)** serum cortisol (μg/dL), **(B)** plasma epinephrine (pg/mL), and **(C)** plasma norepinephrine (pg/mL) at baseline (PRE), immediately post-exercise (POST), and after 30 min of recovery (REC30). Mean values are connected by solid lines to illustrate temporal changes. **p < 0.01, ***p < 0.001 vs. PRE; ^†††^ *p* < 0.001 vs. POST.

Cortisol showed a significant effect of time (F = 13.794, *p* < 0.001). Levels increased from PRE to POST (*p* = 0.008) and from PRE to REC30 (*p* < 0.001), with no difference between POST and REC30 (*p* = 0.530) (Table 3; Figure 1A).

**TABLE 3:** Stress hormones across time points.

Variable	Rest	Immediately post-exercise	30 min recovery	*F*	Post-hoc (Bonferroni)	Partial η^2^
Cortisol (μg/dL)	26.78 ± 3.63	30.39 ± 5.73	31.44 ± 5.75	13.794^a^	PRE < POST, REC30	0.421
Epinephrine (pg/mL)	57.63 ± 11.17	839.57 ± 333.71	68.69 ± 12.81	110.526^a^	PRE < REC30 < POST	0.853
Norepinephrine (pg/mL)	279.39 ± 86.66	4,994.90 ± 1763.09	443.25 ± 102.80	139.744^a^	PRE < REC30 < POST	0.880

Values are mean ± SD; Timepoints: at rest (PRE), immediately post-exercise (POST), and 30 min recovery (REC30).

^a^ *p* < .001.

Epinephrine showed a highly significant time effect (F = 110.526, *p* < 0.001). Concentrations increased at POST compared to those at PRE (*p* < 0.001) and remained elevated at REC30 (*p* = 0.005), although values were significantly lower at REC30 than at POST (*p* < 0.001) (Table 3; Figure 1B).

Norepinephrine also exhibited a robust time effect (F = 139.744, *p* < 0.001). Levels increased significantly from PRE to POST (*p* < 0.001) and from PRE to REC30 (*p* < 0.001), with a decline from POST to REC30 (*p* < 0.001) (Table 3; Figure 1C).

### 3.3 Leukocyte responses

**FIGURE 2:**
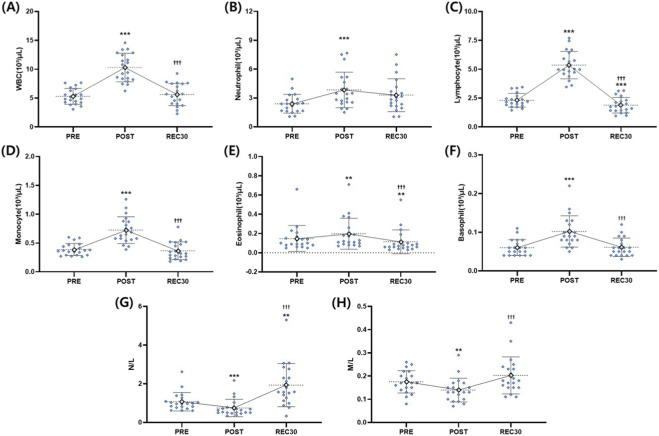
Leukocyte count and differentials across time points. Individual values (blue dots) and group mean ± SD (white diamonds) are displayed for **(A)** total WBC (10^3^/μL), **(B)** neutrophil count (10^3^/μL), **(C)** lymphocyte count (10^3^/μL), **(D)** monocyte count (10^3^/μL), **(E)** eosinophil count (10^3^/μL), **(F)** basophil count (10^3^/μL), **(G)** neutrophil-to-lymphocyte ratio (N/L), and **(H)** monocyte-to-lymphocyte ratio (M/L). Measurements were taken at baseline (PRE), immediately post-exercise (POST), and after 30 min of recovery (REC30). Mean values are connected by solid lines to illustrate temporal trends.**p < 0.01, ***p < 0.001 vs. PRE; ^†††^ *p* < 0.001 vs. POST.

Total white blood cell (WBC) count showed a significant time effect (F = 137.939, *p* < 0.001). WBCs were significantly elevated at POST compared to those at PRE (*p* < 0.001) and returned to baseline at REC30 (*p* = 0.710). Values at POST remained higher than at REC30 (*p* < 0.001) (Table 4; Figure 2A).

**TABLE 4:** Leukocyte count and differentials across time points.

Variable	Rest	Immediately post-exercise	30 min recovery	*F/χ* ^*2*^	Post-hoc (Bonferroni/Wilcoxon)	Partial η^2^
WBC (10^3^/μL)	5.30 ± 1.37	10.29 ± 2.46	5.61 ± 1.92	137.939^c^	PRE, REC30 < POST	0.879
Neutrophil (10^3^/μL)	2.39 ± 0.98	3.84 ± 1.85	3.30 ± 1.72	5.935^a^	PRE, REC30 < POST	0.238
Lymphocyte (10^3^/μL)	2.30 ± 0.59	5.36 ± 1.18	1.87 ± 0.67	291.035^c^	REC30 < PRE < POST	0.939
Monocyte (10^3^/μL)	0.39 ± 0.11	0.72 ± 0.23	0.36 ± 0.16	77.163^c^	PRE, REC30 < POST	0.802
Eosinophil (10^3^/μL)	0.15 ± 0.14	0.20 ± 0.16	0.11 ± 0.12	33.828^c^	REC30 < PRE < POST	0.640
Basophil (10^3^/μL)	0.06 ± 0.02	0.10 ± 0.04	0.06 ± 0.02	48.716^c^	PRE, REC30 < POST	0.719
N/L	1.07 ± 0.48	0.75 ± 0.45	1.93 ± 1.12	χ^2^ = 27.300^c^	PRE > POST^***^; POST < REC30^***^; PRE < REC30^**^	Kendall’s W = 0.683
M/L	0.17 ± 0.05	0.14 ± 0.05	0.20 ± 0.08	13.232^c^	POST < PRE, REC30	0.412

Values are mean ± SD; Timepoints: at rest (PRE), immediately post-exercise (POST), and 30 min recovery (REC30).

^a^ *p* < .05.

^b^ *p* < .01.

^c^ *p* < .001.

Neutrophils showed a significant time effect (F = 5.935, *p* = 0.014). Counts increased significantly at POST relative to those at PRE (*p* < 0.001), with no differences between PRE and REC30 (*p* = 0.111) or between POST and REC30 (*p* = 0.988) (Table 4; Figure 2B).

Lymphocytes showed a pronounced time effect (F = 291.035, *p* < 0.001). Values increased significantly from PRE to POST (*p* < 0.001) but decreased below baseline at REC30 (*p* < 0.001). Furthermore, values at POST were significantly higher than at REC30 (*p* < 0.001) (Table 4; Figure 2C).

Monocytes showed a significant time effect (F = 77.163, *p* < 0.001). Counts were elevated at POST compared to those at PRE (*p* < 0.001) and returned to baseline at REC30 (*p* = 1.000). Values at POST remained significantly higher than at REC30 (*p* < 0.001) (Table 4; Figure 2D).

Eosinophils showed a significant time effect (F = 33.828, *p* < 0.001). Numbers increased at POST relative to those at PRE (*p* = 0.001) but were significantly reduced below baseline at REC30 (*p* = 0.001). Values were significantly higher at POST than at REC30 (*p* < 0.001) (Table 4; Figure 2E).

Basophils showed a significant time effect (F = 48.716, *p* < 0.001). Counts increased at POST compared to those at PRE (*p* < 0.001) and returned to baseline at REC30 (*p* = 1.000). Values at POST were significantly higher than at REC30 (*p* < 0.001) (Table 4; Figure 2F).

The neutrophil-to-lymphocyte ratio (N/L) showed a significant time effect (χ^2^ = 27.300, *p* < 0.001; Friedman test). The ratio decreased significantly from PRE to POST (*p* < 0.001) and increased markedly at REC30 compared to POST (*p* < 0.001). Values at REC30 were also significantly higher than those at PRE (*p* = 0.002) (Table 4; Figure 2G).

The monocyte-to-lymphocyte ratio (M/L) showed a significant time effect (F = 13.232, *p* < 0.001). Values decreased significantly from PRE to POST (*p* = 0.005), trended toward baseline at REC30 (*p* = 0.195), and were significantly higher at REC30 than at POST (*p* < 0.001) (Table 4; Figure 2H).

### 3.4 Muscle damage markers

**FIGURE 3:**
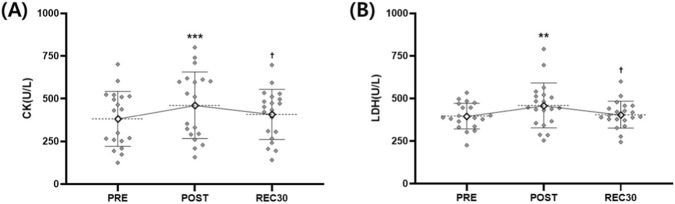
Muscle damage markers (CK, LDH) across time points. Individual values (grey diamonds) and group mean ± SD (white diamonds) are shown for **(A)** creatine kinase (CK, U/L) and **(B)** lactate dehydrogenase (LDH, U/L) at baseline (PRE), immediately post-exercise (POST), and after 30 min of recovery (REC30). Mean values are connected with solid lines to illustrate temporal changes. **p < 0.01, ***p < 0.001 vs. PRE; ^†^ *p* < 0.05 vs. POST.

CK showed a significant time effect (F = 18.966, *p* < 0.001). Levels increased significantly from PRE to POST (*p* < 0.001), but no differences were observed between PRE and REC30 (*p* = 0.074). However, values at POST were higher than at REC30 (*p* = 0.019) (Table 5; Figure 3A).

**TABLE 5:** Muscle damage markers (CK, LDH) across time points.

Variable	Rest	Immediately post-exercise	30 min recovery	*F*	Post-hoc (Bonferroni)	Partial η^2^
CK (U/L)	381.65 ± 160.60	461.20 ± 194.95	407.90 ± 146.86	18.966^a^	PRE, REC30 < POST	0.500
LDH (U/L)	397.05 ± 75.58	459.25 ± 131.92	405.15 ± 79.41	10.112^b^	PRE, REC30 < POST	0.347

Values are mean ± SD; Timepoints: at rest (PRE), immediately post-exercise (POST), and 30 min recovery (REC30).

a *p* < .001.

^b^ *p* < .01.

Lactate dehydrogenase (LDH) showed a significant time effect (F = 10.112, *p* = 0.005). Values increased significantly at POST compared to those at PRE (*p* = 0.007), but no differences were found between PRE and REC30 (*p* = 0.864). LDH values were significantly higher at POST than at REC30 (*p* = 0.019) (Table 5; Figure 3B).

## 4 Discussion

The present study characterizes acute physiological responses to a maximal 2,000-m rowing ergometer trial in elite male rowers, focusing on stress hormones, leukocyte subsets, and muscle damage markers. The results indicate that high-intensity rowing imposes substantial neuroendocrine, immunological, and musculoskeletal stress, providing insight into athlete monitoring during heavy training or competition periods.

Cortisol increased significantly from pre-exercise to post-exercise and remained elevated 30 min into recovery, reflecting activation of the HPA axis in response to metabolic and mechanical stress (Pedersen and Hoffman-Goetz, 2000). This acute elevation facilitates mobilization of energy substrates and modulation of inflammatory pathways but may compromise recovery if sustained chronically (Haller et al., 2023). The absence of a non-exercise control session may be viewed as a limitation. However, acute steroid hormone responses, particularly cortisol have been shown to exhibit high test–retest reliability following short-duration high-intensity exercise (Leal et al., 2019). Epinephrine and norepinephrine also increased post-exercise, followed by partial recovery at 30 min, consistent with rapid sympathetic-adrenal-medullary activation during high-intensity exercise (Pedersen and Hoffman-Goetz, 2000). These patterns align with prior rowing studies and underscore the need to consider both immediate and residual neuroendocrine stress when planning successive training sessions (Gomes et al., 2020).

Neutrophil counts increased immediately after exercise but returned to baseline within 30 min of recovery, indicating that the post-exercise neutrophilia was transient rather than sustained (Sellami et al., 2018; Markov et al., 2023). Neutrophils increased post-exercise and remained elevated, whereas lymphocytes increased immediately after exercise but declined below baseline at recovery, indicating a temporary lymphocytopenia and transient immunosuppression (Pedersen and Hoffman-Goetz, 2000; Markov et al., 2023). Although a transient decrease in lymphocyte counts has traditionally been described as an “open window” of immune vulnerability, recent literature suggests that this phenomenon is more complex and may vary depending on exercise intensity, recovery status, and individual training load (Sellami et al., 2018). Therefore, while our findings show a short-term lymphocyte reduction, this does not necessarily equate to clinically meaningful immunosuppression, and the interpretation should be made with caution.

Both the neutrophil-to-lymphocyte (N/L) and monocyte-to-lymphocyte (M/L) ratios exhibited distinct kinetic patterns, characterized by an immediate post-exercise decline followed by a rebound above baseline during early recovery. These changes were primarily driven by a marked reduction in circulating lymphocytes—likely reflecting stress-induced lymphocyte trafficking into peripheral tissues—combined with the delayed mobilization of neutrophils and monocytes back into the circulation (Shek et al., 1995). The overshoot of N/L and M/L at REC30 suggests a transient shift toward innate immune dominance and a mild pro-inflammatory bias during early recovery. This dynamic interplay between lymphocyte redistribution and innate cell mobilization highlights the complexity of post-exercise immune regulation and supports the view that these ratios reflect coordinated immune functional reorganization rather than uniform suppression.

Although post-exercise lymphocytopenia and increases in N/L or M/L ratios have often been interpreted as transient immune suppression, emerging evidence indicates that these responses may represent an adaptive redistribution of immune cells. Activated T cells, γδ T lymphocytes, and NK cells are known to migrate from the bloodstream to peripheral tissues following acute exercise, supporting immune surveillance and tissue repair rather than reflecting pathological suppression (Anane et al., 2009; Dhabhar, 2014; Walsh, 2018).

Consistent with this perspective, high-intensity exercise has been shown to reduce neutrophil phagocytic capacity while increasing γδ T-cell proportions (Leal et al., 2021), suggesting functional reorganization of the immune system. Therefore, the 30-min post-exercise immune pattern in this study may reflect a normal and adaptive stress response. However, repeated exposure without adequate recovery may still increase infection risk due to cumulative immune strain, particularly in high-performance settings.

CK and LDH increased significantly post-exercise, consistent with sarcolemmal disruption and metabolic stress associated with repeated high-intensity concentric and eccentric contractions of large muscle groups (Gomes et al., 2020; Haller et al., 2023). Although levels partially recovered at 30 min, the post-exercise elevations indicate that a single 2,000-m session imposes a meaningful mechanical load capable of inducing transient subclinical muscle stress, rather than a persistent muscle damage. These findings support integrating recovery modalities such as active recovery, nutrition, and periodized training to mitigate overuse injuries in elite rowers (Gomes et al., 2020; Chang et al., 2024).

Concurrent elevations in stress hormones, leukocyte redistribution, and muscle damage markers illustrate multifactorial stress during a 2,000-m effort. The temporal dissociation between neuroendocrine recovery (partial at 30 min) and immune modulation (lymphocyte nadir) suggests that physiological restoration is not uniform across systems. Coaches should account for these temporal differences in CK and LDH responses—post-exercise elevations and their return toward baseline at 30 min—when prescribing training frequency and intensity, to avoid cumulative stress that could compromise performance or precipitate overtraining (Pedersen and Hoffman-Goetz, 2000; Haller et al., 2023).

The observed biomarker patterns provide a rationale for individualized monitoring. Compared with studies using longer recovery periods, which reported persistent elevations in CK and cortisol for several hours post-exercise (e.g., Haller et al., 2023; Pedersen and Hoffman-Goetz, 2000), our results show rapid partial recovery by 30 min. Athletes displaying exaggerated cortisol or CK responses might benefit from extended recovery, whereas those with blunted leukocyte responses may require adjustments in intensity or volume to prevent immunosuppression. This integrative biomarker approach aligns with precision exercise physiology principles aimed at optimizing performance while minimizing injury and illness risk (Markov et al., 2023).

This study has some limitations. First, responses were assessed at only a single recovery time point (30 min). The short recovery window was chosen to capture immediate post-exercise physiological responses relevant to acute training stress. However, markers such as CK and cortisol may continue to fluctuate over several hours, and their full return to baseline could influence subsequent training sessions. Future studies should include multiple post-exercise time points to better define the temporal trajectory of hormonal, immunological, and muscle damage markers. Second, the sample was restricted to elite male rowers, which limits the generalizability of our findings. Inclusion of female athletes and individuals at different competitive levels would be necessary to determine whether these biomarker responses are consistent across broader populations.

## 5 Conclusion

This study highlights the acute physiological demands of a maximal 2,000-m rowing ergometer trial, revealing integrated neuroendocrine, immune, and musculoskeletal responses. Substantial elevations in cortisol and catecholamines confirm intense metabolic and sympathetic activation, while transient lymphocytopenia and dynamic shifts in N/L and M/L ratios reflect coordinated post-exercise immune reorganization rather than sustained immunosuppression. Elevated CK and LDH indicate mechanical and metabolic strain, providing objective markers of exercise-induced musculoskeletal stress.

These findings underscore that elite rowing is a multifactorial challenge requiring simultaneous optimization of cardiovascular, neuromuscular, and immune systems. Differential recovery kinetics across systems highlight the importance of individualized monitoring and periodized recovery. From a practical perspective, future interventions—such as nutritional, pharmacological, or active recovery strategies—could help modulate stress responses and enhance recovery. Overall, understanding these acute responses provides an evidence-based framework for designing training, recovery, and injury-prevention strategies, offering actionable insights for coaches, sports scientists, and exercise physiologists aiming to optimize performance and safeguard athlete health.

## Data Availability

The raw data supporting the conclusions of this article will be made available by the authors, without undue reservation.
