# Correction: Acute endocrine, immune, and muscle damage responses following a 2,000-m rowing time trial in elite male athletes

**DOI:** 10.3389/fphys.2025.1771138

**Published:** 2026-01-08

**Authors:** Soo-Min Ha, Min-Seong Ha, Minchul Lee

**Affiliations:** 1 Department of Physical Education, Pusan National University, Busan, Republic of Korea; 2 Laboratory of Sports Conditioning: Nutrition, Biochemistry, and Neuroscience, Department of Sport Science, College of Arts and Sports, University of Seoul, Seoul, Republic of Korea; 3 Department of Sports Medicine, College of Health Science, CHA University, Pocheon-si, Gyeonggi-do, Republic of Korea

**Keywords:** rowing, stress hormones, immune response, muscle damage, recovery, elite atletes

The grant from the National Research Foundation of Korea (Grant number: NRF-2023S1A5A2A01079293) was erroneously omitted. The Research Grant (Grant No. KSSO202502) from the Korean Society for the Study of Obesity to Minchul Lee was erroneously omitted. The Basic Study and Interdisciplinary R&D Foundation Fund of the University of Seoul (2025) to Min-Seong Ha was erroneously omitted.

The correct Funding statement is: “The author(s) declare that financial support was received for the research and/or publication of this article. This study was supported by the National Research Foundation of Korea (Grant number: NRF-2023S1A5A2A01079293) and by a Research Grant (Grant No. KSSO202502) from the Korean Society for the Study of Obesity by Minchul Lee. Also this work was supported by the Basic Study and Interdisciplinary R&D Foundation Fund of the University of Seoul (2025) by Min-Seong Ha.”

The original version of this article has been updated.

